# Association Between Older Age and TIPS-Related Hospitalization Following Shunt Placement

**DOI:** 10.1155/cjgh/8894058

**Published:** 2025-05-21

**Authors:** Matthew Schliep, Brian J. Wentworth, Indira Bhavsar-Burke, Anthony Rainho, Megha Chiruvella, Matthew J. Stotts, Marwan Ghabril

**Affiliations:** ^1^Department of Medicine, University of Virginia, Charlottesville, Virginia, USA; ^2^Division of Digestive and Liver Disease, UT Southwestern, Dallas, Texas, USA; ^3^Division of Gastroenterology and Hepatology, Department of Medicine, University of Virginia, Charlottesville, Virginia, USA; ^4^Division of Gastroenterology and Hepatology, Department of Medicine, Indiana University, Indianapolis, Indiana, USA

**Keywords:** chronic liver disease, cirrhosis, portal hypertension, transjugular intrahepatic portosystemic shunt

## Abstract

**Background and Aims:** Patients experience more complications of portal hypertension as liver disease progresses, many of which can be managed by transjugular intrahepatic portosystemic shunt (TIPS) insertion. Controversy surrounds the association of age with TIPS-related complications. We sought to evaluate the effect of age on TIPS-associated outcomes, including hospital admissions.

**Methods:** This retrospective, bicentric cohort study included patients who underwent TIPS insertion between January 1, 2006, and December 31, 2016. The primary outcome of the study was predictors of liver-related hospital admission within 12 months of TIPS insertion between patients < 70 years and ≥ 70 years old. Secondary outcomes included mortality at 12 months and MELD-Na score at 90 days following TIPS placement.

**Results:** A total of 593 patients were included in the study—487 patients were less than 70 years old while 106 patients were 70 years of age or older. Near equal percentages of elderly and nonelderly patients were admitted with post-TIPS complications within 12 months of insertion (29.2% v. 29.0%, *p*=0.91). Pre-existing diagnoses of diabetes and/or hypertension, hepatic hydrothorax, as well as serum creatinine and/or serum sodium at the time of TIPS insertion were associated with TIPS-related admissions within the first 12 months of shunt insertion.

**Conclusion:** TIPS placement in selected older patients can be safe. Age should not be a strict contraindication for TIPS insertion, but discussion regarding risks and benefits of the procedure should be individualized.

## 1. Introduction

Chronic liver disease (CLD) is the most common cause of portal hypertension (PH) in the United States [1]. Clinically significant portal hypertension (CSPH), which occurs at a hepatic venous pressure gradient (HVPG) of 10 mmHg or greater, is associated with an increased risk of decompensated liver disease and its associated complications, characterized by ascites, variceal hemorrhage (VH), and/or hepatic encephalopathy (HE) [2].

Decompression of the portal system by transjugular intrahepatic portosystemic shunt (TIPS) insertion effectively treats these complications; it is also associated with improved pretransplant mortality [3]. Technical advancements over time have expanded indications for TIPS and candidacy for the procedure to greater numbers of patients [4]. Placement of a TIPS, however, is not without risk. Common complications of TIPS insertion include HE, shunt stenosis, or heart failure, while less frequent complications include endotipsitis and hemorrhage [3].

Numerous risk factors have been proposed when evaluating patients for TIPS. These include prior history of HE, cardiopulmonary status, and frailty [5]. Controversy surrounds the association of age with post-TIPS complications and outcomes, with many providers avoiding TIPS in older patients given previously unexplored postprocedural risks in this patient population. In clinical practice, tools such as the Model for End-stage Liver Disease (MELD) score are used to risk-stratify patients for TIPS placement and predict transplant-free survival following TIPS insertion [6].

Overall, there is limited age-related guidance for cirrhosis care in older patients despite an increase in the number of older patients with cirrhosis [7]. The newly developed Freiburg Index for Post-TIPS survival (FIPS) is one of the few models that incorporate age, in addition to serum creatinine, serum bilirubin, and serum albumin for prognostication, though its use is yet to be widely adopted [8, 9]. Prior work has indicated that a higher prevalence of medical comorbidities and increased frailty in the elderly population may limit treatment options for CLD even beyond considerations of liver transplantation [7].

Most data regarding the safety of TIPS in the elderly are derived from smaller and/or single-center observational studies; results of these studies are inconsistent. Prior work by Parvinian et al. found age to be a predictor of early mortality post-TIPS [10]. Other studies have suggested that age is not associated with mortality at 30 days and 1 year following TIPS insertion but is associated with an increased rate of complications, including hospitalizations [11, 12]. The recent work aimed at developing and validating a prognostic model for TIPS insertion, specifically in the elderly population, identified serum creatinine and sodium levels as independent predictors of post-TIPS mortality, but data evaluating associations between age and TIPS insertion with hospital admissions remain limited [4]. Given the uncertainty surrounding risk of TIPS and TIPS-related complications in the elderly population, we sought to evaluate the relationship between age and TIPS-related hospitalizations and outcomes in a bicenter study.

## 2. Methods

### 2.1. Study Design

This retrospective, multicenter cohort study included patients ≥ 18 years who underwent TIPS placement with a covered stent between January 1, 2006, and December 31, 2016. The study was approved by the Institutional Review Board at participating sites, and data share agreements were completed (University of Virginia (UVA), Charlottesville, VA; Indiana University (IU), Indianapolis, IN).

### 2.2. Data Collection and Study Population

Potential patients were identified by having the current procedural terminology (CPT) code for TIPS insertion (37,182) in addition to one/more of the following International Classification of Diseases, 10th revision (ICD, or equivalent 9th revision) codes at hospital admission and/or discharge ascribed to cirrhosis or its complications as previously reported: I85.00, I85.01, I85.10, I85.11, K72.10, K72.11, K72.90, K72.91, K76.6, or R18.8 [13]. Patients were excluded from the study for age less than 18, previous liver transplantation, or absence of cirrhosis on chart review. Demographics data, medical comorbidities, baseline characteristics, portal pressure measurements before and after TIPS placement, and clinical outcomes of interest were collected via chart review. Due to differences in reporting parameters between the two sites, true HVPGs were inconsistently measured during TIPS insertion. Thus, portosystemic gradient was reported as a surrogate (defined as the difference in the wedged hepatic pressure and the right atrial pressure).

### 2.3. Outcomes

The primary outcome of the study was TIPS-related hospitalization within 12 months of TIPS insertion. Reasons for hospitalization were considered TIPS-related if patients were admitted for HE, shunt dysfunction or stenosis, or cardiac dysfunction. Patients who underwent liver transplantation and had subsequent hospital admissions were not included and considered to have TIPS-related hospital admissions. Secondary outcomes of the study included change in MELD-Na score at 90-day post-TIPS, hospital readmissions for non-TIPS related reasons within 12 months, and mortality at 90 days and 12 months.

### 2.4. Statistical Analysis

Descriptive statistics utilized two-sided Student's *t*-tests for continuous variables and X^2^ test for categorical variables. Continuous variables are presented as mean ± SD; categorical variables are described as counts and percentages. Patients were stratified into dichotomous subcohorts by age (< 70 years and ≥ 70 years) in accordance with prior literature [4]. Univariate linear regression with age as a covariate was conducted with significant predictors (*p* < 0.10) imputed into a forward stepwise multivariate regression model. Cox proportional hazards analysis was used to identify multivariable prognostic factors for TIPS-associated readmissions using statistically significant variables between age cohorts and others identified as being clinically relevant. The Kaplan–Meier method was used to assess the probability of mortality. All statistical analysis was performed using R Statistical Software, Version 4.1.2 (R Core Team, Vienna, Austria).

## 3. Results

### 3.1. Patient Characteristics

A total of 593 patients meeting inclusion criteria were identified between the two sites—487 patients were less than 70 years old while 106 patients were 70 years or older (Supporting Figure 1). The average age of patients less than 70 was 54.0 ± 8.2 years, and the average age of patients aged 70 and older was 74.3 ± 4.6 years. There were significantly more female patients in the elderly cohort. Alcohol was the predominant etiology of cirrhosis (42.9%) in the younger cohort, while nonalcoholic steatohepatitis (NASH) was most common in the elderly cohort (38.7%). There were statistically significant differences in medical comorbidities, such as coronary artery disease and hypertension, in the elderly cohort compared to the nonelderly cohort. Demographics data and baseline characteristics are depicted in Table 1.

There was no difference between age groups in the severity of CLD, measured by MELD-Na, at the time of TIPS placement (Table 1). The average MELD-Na score at the time of TIPS placement was 15.9 ± 6.8 in the younger cohort and 15.3 ± 4.8 in the older cohort. There were similar rates of severity of pre-TIPS HE (mild HE defined as precipitant induced or well-controlled and severe defined by one or more hospital admissions despite dual therapy) and ascites (severe noted as “diuretic refractory” on chart review) in both cohorts. Pre- and post-TIPS PSG were not different between younger and older patients (mean pre-TIPS: 17.7 mmHg vs. 17.2 mmHg, *p*=0.360; post-TIPS: 7.9 mmHg vs. 7.3 mmHg, *p*=0.860). There was no difference in TIPS diameter between groups. The average MELD-Na at 90 days following shunt insertion was 16.0 ± 6.3 in the younger cohort (baseline 15.9 ± 6.8) and 16.4 ± 7.7 in the older cohort (baseline 15.3 ± 4.8).

### 3.2. Post-TIPS Outcomes

TIPS-associated readmissions, including HE, shunt dysfunction, and shunt stenosis, occurred in approximately 29% of patients in each age cohort, as depicted in Table 2 (*p*=0.905). There was no difference in hospitalizations for any reason between groups (*p*=0.879). Readmission for post-TIPS HE occurred in 12.3% and 18.9% of younger and older patients, respectively (*p*=0.074). Significantly more patients in the older group had multiple TIPS-associated hospital readmissions (5.6% vs. 30.2%, *p*=0.040).

**Figure 1:**
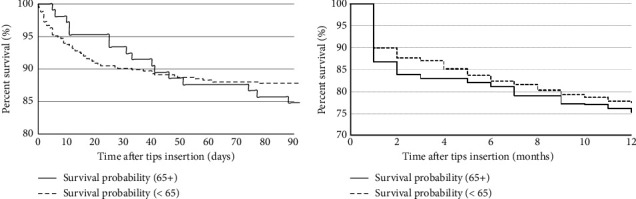
Kaplan–Meier survival curve of patients at 90 days and 12 months following TIPS insertion. 12-month mortality data were available on 49.8% of patients, including 239 patients < 70 years old and 56 patients ≥ 70 years old. Patients were censored if lost to follow-up; liver transplantation was considered a competing event.

On Cox proportional regression analysis, pre-existing diagnoses of diabetes and/or hypertension, hepatic hydrothorax, as well as serum creatinine and/or serum sodium at the time of TIPS insertion were associated with TIPS-related admissions within the first 12 months of shunt insertion (Table 3). Age was not a significant predictor of hospital admission following TIPS placement (0.98). Kaplan–Meier analysis stratified by age cohort showed no difference between groups in liver-related mortality at 90 days or 12 months (*p*=0.387 and *p*=0.406, respectively) (Figure 1).

## 4. Discussion

As the population ages, TIPS continues to be used as a destination therapy in addition to a bridge to liver transplantation [14]. Despite ongoing controversy in the existing literature regarding the role of TIPS in the elderly, our study adds to a growing body of evidence suggesting that TIPS is safe in this population. We identified serum sodium at the time of TIPS insertion to be an independent risk factor for post-TIPS hospital admission in elderly patients, in line with recently published data suggesting that it is also associated with post-TIPS mortality in a similar population [4]. While other studies have reached similar conclusions on age and mortality post-TIPS, there are notable differences between the age cutoffs (reported in other studies as either ≥ 65 years or ≥ 75 years), sample sizes, and methodology (specifically regarding shunt size and type) that make drawing conclusions for clinical practice difficult [11, 15–17].

Despite there being no consensus regarding the association of age and TIPS-related outcomes, there does seem to be consistent evidence that older age is associated with more post-TIPS complications, including higher rates of HE and more frequent hospitalizations [18, 19]. Our study builds on this theme. While there was no difference between groups regarding percentage of patients requiring hospitalization for TIPS-related complications, older age was associated with multiple TIPS-related hospitalizations within 12 months of shunt insertion. Furthermore, we demonstrated a trend toward increased admissions for post-TIPS HE though there was no statistical difference between age cohorts in our study.

These findings explain the repeated attempts to further elucidate the association of age and mortality following TIPS placement. Recent practice guidance from the Advancing Liver Therapeutic Approaches consensus reports insufficient evidence for recommending age cutoffs for TIPS insertion [20]. When prognostication tools, such as FIPS, incorporate age for risk stratification, they do not fare better than those without age in their calculation, such as the MELD-Na and Child–Pugh scores [9, 21, 22].

Perhaps a more specific measure of patient metabolic age, rather than the chronologic age, would further clarify these findings. Recent data regarding the roles of sarcopenia and frailty in outcomes for patients with end-stage liver disease have evaluated this concept. Works by Ronald et al. and Praktiknjo et al. have demonstrated that sarcopenia is associated with increased mortality following TIPS insertion [23, 24]. As the understanding of aging and liver disease improves, revision of existing or derivation of new prognostic scoring systems for post-TIPS complications are needed. Given the available evidence which is further supported by the results of our study, older patients considering TIPS warrant personalized counseling regarding potential challenges following the procedure, including discussion regarding the risk of hospitalization and development of post-TIPS HE.

There are limitations to our study that warrant discussion including its retrospective design, surrogate use of PSG for HVPG given inconsistent reporting and documentation between proceduralists and across institutions, and our inability to capture concurrent medication use that could precipitate HE. Interpretation of our findings is limited by selection bias; patients who have undergone TIPS insertion, especially in the elderly population, likely have better baseline functional status compared to patients who did not undergo TIPS insertion. Our study was also not powered to detect differences in age cohorts at specific cutoffs (such as just those patients ≥ 75 years), though there is no accepted standard on what constitutes “older” age for TIPS [10, 12, 22, 25–28]. Furthermore, each participating center in our study has notable multidisciplinary expertise in hepatology and interventional radiology; variations in medical care and technical skill could limit the generalizability of our results beyond similar centers.

To the best of our knowledge, our bicenter cohort includes both the highest total number of patients among studies evaluating the association between age and, specifically, TIPS-related hospitalizations. Both cohorts had similar baseline characteristics, such as MELD-Na score, TIPS shunt diameter, and pre- and post-TIPS HVPG improving the integrity of our results. All patients included in the study received fully covered stents, and we did not exclude patients based on indication for TIPS placement.

While liver transplantation remains the definitive therapy for end-stage liver disease, TIPS improves survival and quality of life among patients awaiting transplant [3, 14, 29]. As the population of patients' ages, our study indicates that TIPS in selected older patients is safe. Older patients may not be eligible for liver transplantation, and TIPS may be an important consideration for controlling complications of cirrhosis. Therefore, age should not be a contraindication to TIPS insertion, but careful discussion regarding the risks and benefits of the procedure needs to be individualized for each patient. Future studies may consider the effect of the metabolic age or disease severity, measured by liver frailty index or measures of sarcopenia, on outcomes following TIPS placement.

## Figures and Tables

**Table 1:** Demographics data and characteristics of patients by age cohort, displayed as means (SD) or counts (percentages).

Clinical and demographic variables by age cohort	Patients < 70 (*N* = 487)	Patients ≥ 70 (*N* = 106)	*p* value
Age	54.0 (8.2)	74.3 (4.6)	**< 0.001**
Sex			
Male	331 (68.0)	60 (56.6)	**< 0.001**
Female	156 (32.0)	46 (43.3)	
Race			
White	430 (88.3)	98 (92.5)	0.12
Black	41 (8.4)	7 (6.6)	
Others	16 (3.3)	1 (0.9)	
Cirrhosis etiology			
Alcohol related	209 (42.9)	24 (22.6)	**< 0.001**
Nonalcohol steatohepatitis	83 (17.0)	41 (38.7)	
Viral hepatitis	121 (24.8)	6 (5.7)	
Others	74 (15.2)	35 (33.0)	
Diabetes mellitus	169 (34.7)	48 (45.3)	**0.003**
Tobacco use	210 (43.1)	29 (27.4)	**< 0.001**
Hypertension	145 (29.8)	49 (46.2)	**< 0.001**
Coronary artery disease	37 (7.6)	16 (15.1)	**< 0.001**
Chronic kidney disease	170 (34.9)	37 (38.5)	0.794
Na (meq/mL)	134.3 (5.4)	135.1 (4.8)	0.135
SCr (mg/dL)	1.2 (0.6)	1.3 (0.9)	**0.042**
Albumin (g/dL)	3.0 (0.6)	3.1 (0.6)	0.552
TB (mg/dL)	2.4 (3.4)	1.9 (1.8)	0.080
INR	1.4 (0.4)	1.3 (0.2)	**0.041**
MELD-Na at TIPS insertion	15.9 (6.8)	15.3 (4.8)	0.098
HE present	179 (36.8)	31 (29.2)	0.092
HE severity			
Mild	169 (34.7)	28 (26.4)	
Severe	10 (2.1)	3 (2.8)	
Ascites present	386 (79.3)	78 (73.6)	0.275
Ascites severity			
Mild/moderate	83 (17.0)	19 (17.9)	
Refractory	304 (62.4)	59 (55.7)	
Pre-PSG (mmHg)	17.7 (5.8)	17.2 (4.9)	0.360
Post-PSG (mmHg)	7.9 (3.2)	7.3 (2.9)	0.860
TIPS shunt diameter (mm)			
6	1 (0.2)	0 (0.0)	0.780
7	4 (0.8)	4 (3.8)	
8	291 (59.8)	68 (64.2)	
9	57 (11.7)	9 (8.5)	
10	121 (24.8)	22 (20.8)	
12	4 (0.8)	1 (2.8)	
Indications for TIPS			
Ascites	386 (79.3)	78 (73.6)	0.090
VH	61 (12.5)	12 (11.3)	
Hepatic hydrothorax	40 (8.2)	16 (15.1)	

*Note:* ALD, alcohol-related liver disease; CAD, documented history of coronary artery disease at the time of TIPS insertion; CKD, documented history of chronic kidney disease at the time of TIPS insertion; DM, documented history of diabetes mellitus at the time of TIPS insertion; HE, documented history of hepatic encephalopathy at the time of TIPS insertion; HTN, documented history of hypertension at the time of TIPS insertion; MELD, Model End-Stage Liver Disease; NASH, nonalcoholic steatohepatitis. Bold values are statistically significant with a *p* value < 0.05.

Abbreviations: AVB, acute variceal bleeding; CTP, Child–Turcotte–Pugh; HCV, hepatitis C virus; HVPG, hepatic venous pressure gradient; TIPS, transjugular intrahepatic portosystemic shunt.

**Table 2:** TIPS-related outcomes compared between age cohorts.

TIPS-related outcomes by age cohort	Patients < 70 years (*N* = 487)	Patients ≥ 70 years (*N* = 106)	*p* value
Hospitalized within 12 months (all admissions, liver- and non–liver-related, planned or otherwise)	256 (52.6)	51 (48.1)	0.88
TIPS-associated admissions within 12 months	141 (29.0)	31 (29.2)	0.91
Multiple TIPS-associated readmission within 12 months	27 (5.6)	32 (30.2)	**0.04**
Reason for TIPS-associated admission			
HE	60 (12.3)	20 (18.9)	0.14
Shunt dysfunction	23 (4.7)	1 (0.9)	
Shunt stenosis	16 (3.3)	2 (1.9)	
Cardiac dysfunction	7 (1.4)	1 (0.9)	

*Note:* Data are shown as mean (standard deviation) or number (percentage). Bold value is statistically significant with a *p*-value < 0.05.

Abbreviations: HE, hepatic encephalopathy; TIPS, transjugular intrahepatic portosystemic shunt.

**Table 3:** Prognostic factors associated with TIPS-related hospital admissions.

Clinical and demographic variables of interest	Coeff.	CI	*p* value
Age	0.01	(0.75; 1.68)	0.98
CAD	3.15	(6.25; 7.23)	0.97
DM	6.90	(6.52; 7.61)	**< 0.01**
HTN	9.71	(1.07; 8.81)	**< 0.01**
MELD-Na	7.79	(5.53; 11.09)	0.09
Pre-HVPG	1.26	(2.19; 7.27)	0.08
Post-HVPG	6.67	(1.65; 2.70)	0.05
Reason for TIPS (hepatic hydrothorax)	0.10	(2.69; 3.37)	**< 0.01**
Serum creatinine	1.22	(3.21; 4.59)	**< 0.01**
Serum sodium	1.24	(1.91; 8.10)	**< 0.01**
Sex	0.03	(1.10; 9.10)	0.87
Tobacco use	1.01	(1.17; 9.10)	0.99

*Note:* CAD, documented history of coronary artery disease at the time of TIPS insertion; DM, documented history of diabetes mellitus at the time of TIPS insertion; HTN, documented history of hypertension at the time of TIPS insertion; MELD, Model End-Stage Liver Disease. Bold values are statistically significant with a *p* value < 0.05.

Abbreviations: HVPG, hepatic venous pressure gradient; TIPS, transjugular intrahepatic portosystemic shunt.

## Data Availability

Data and study materials may be available upon request.

## Supporting Information

Supporting data include baseline pre-TIPS characteristics and demographics compared in patients between clinical sites and patient flow diagram.
