# Supplementary material for: Association Between Older Age and TIPS-Related Hospitalization Following Shunt Placement
Source: Can J Gastroenterol Hepatol. 2025 May 21;2025:8894058. doi: 10.1155/cjgh/8894058 (PMC12119167; doi:10.1155/cjgh/8894058)
Supplement: Supporting Information — Additional supporting information can be found online in the Supporting Information section. [file 8894058.f1.docx]

**Supplementary Figure 1**: Patient flow diagram.


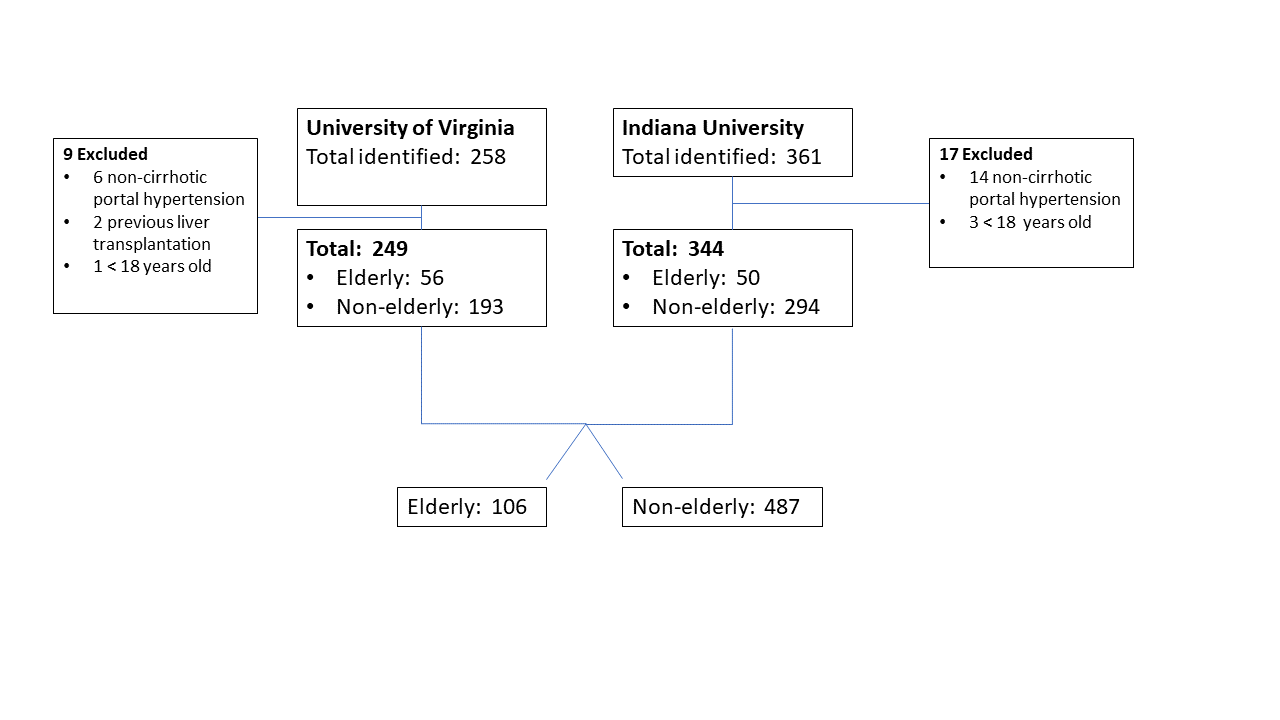


**Supplementary Table 1:** Baseline pre-TIPS characteristics and demographics compared in patients between clinical sites. Data are shown as mean (standard deviation) or number (percentage).

| Clinical and demographic variables by site | IU  (N = 344) | IU patients < 70 (N = 294) | IU patients ≥ 70 (N = 50) | UVA  (N = 249) | UVA patients < 70 (N = 193) | UVA patients ≥ 70 (N = 56) |
| --- | --- | --- | --- | --- | --- | --- |
| Age | 55.0 (8.8) | 53.9 (7.4) | 73.8 (3.4) | 56.4 (11.0) | 54.5 (8.6) | 72.9 (4.9) |
| Sex  Male    Female | 247 (71.7)  97 (28.3) | 213 (72.3)  81 (27.7) | 34 (68.0)  16 (32.0) | 156 (62.6)  93 (37.4) | 109 (56.4)  84 (48.8) | 47 (84.0)  9 (16.0) |
| Race  White    Black    Other | 291 (84.5)  44 (12.8)  9 (2.7) | 245 (83.2)  40 (13.6)  9 (3.2) | 46 (92.0)  4 (8.0)  0 | 228 (91.7)  13 (5.3)  8 (3.0) | 178 (91.5)  9 (4.9)  6 (3.6) | 50 (89.3)  4 (7.1)  2 (3.6) |
| Cirrhosis etiology  ALD    NASH    Viral hepatitis    Other | 146 (42.5)  64 (18.7)  107 (31.1)  27 (7.7) | 131 (44.5)  43 (14.7)  100 (34.0)  20 (6.8) | 15 (30.0)  21 (42.0)  7 (14.0)  7 (14.0) | 93 (37.4)  55 (22.2)  39 (15.7)  62 (25.0) | 81 (42.0)  36 (18.4)  37 (19.1)  39 (20.5) | 12 (21.4)  19 (33.9)  2 (3.6)  23 (41.1) |
| Diabetes Mellitus | 114 (33.2) | 92 (31.4) | 22 (44.0) | 109 (43.6) | 76 (39.6) | 33 (58.9) |
| Tobacco use | 128 (37.1) | 117 (39.7) | 11 (22.2) | 111 (44.4) | 93 (48.1) | 18 (32.1) |
| Hypertension | 77 (22.4) | 51 (17.2) | 26 (52.0) | 103 (41.4) | 74 (38.2) | 29 (51.8) |
| Coronary artery disease | 31 (9.1) | 14 (4.7) | 16 (32.0) | 30 (12.0) | 20 (10.6) | 10 (17.9) |
| Chronic kidney disease | 148 (43.1) | 106 (36.2) | 42 (84.0) | 36 (14.4) | 25 (13.1) | 11 (19.6) |
| Indication for TIPS  Ascites    VH    Hepatic hydrothorax | 277 (80.6)  59 (17.2)  8 (2.2) | 235 (79.9)  55 (18.6)  4 (1.5) | 42 (84.0)  4 (8.0)  4 (8.0) | 191 (76.7)  23 (9.1)  35 (14.2) | 152 (78.8)  16 (8.1)  25 (13.1) | 36 (69.6)  7 (12.85  13 (23.2) |
| Na (meq/mL) | 133.3 (5.4) | 133.1 (5.5) | 135.1 (4.3) | 135.1 (5.1) | 135.1 (5.1) | 135.0 (5.0) |
| SCr (mg/dL) | 1.3 (0.7) | 1.2 (0.7) | 1.5 (0.8) | 1.2 (0.7) | 1.2 (0.6) | 1.3 (0.9) |
| Albumin (g/dL) | 2.9 (0.6) | 2.9 (0.6) | 3.1 (0.4) | 3.1 (0.6) | 3.1 (0.6) | 3.2 (0.6) |
| TB (mg/dL) | 2.7 (4.4) | 2.8 (4.6) | 2.0 (2.1) | 2.1 (2.2) | 2.1 (2.4) | 1.9 (1.7) |
| INR | 1.4 (0.4) | 1.5 (0.4) | 1.2 (0.2) | 1.4 (0.4) | 1.4 (0.4) | 1.4 (0.3) |
| MELD-Na | 15.6 (5.9) | 15.7 (6.4) | 15.3 (6.9) | 15.7 (6.3) | 15.7 (6.4) | 15.6 (4.0) |
| Pre-PSG (mmHg) | 16.1 (4.3) | 16.0 (4.2) | 16.5 (4.8) | 17.6 (5.6) | 17.7 (5.8) | 17.3 (4.9) |
| Post-PSG (mmHg) | 6.2 (2.1) | 6.2 (2.1) | 5.6 (2.0) | 7.7 (3.1) | 7.9 (3.2) | 7.3 (2.9) |
